# Photobiotechnology for abiotic stress resilient crops: Recent advances and prospects

**DOI:** 10.1016/j.heliyon.2023.e20158

**Published:** 2023-09-15

**Authors:** Mayank Anand Gururani

**Affiliations:** Biology Department, College of Science, UAE University, Al Ain, United Arab Emirates

**Keywords:** *Arabidopsis*, Abiotic stress, Mutant, Phytochrome, Stress tolerance, Transgenic

## Abstract

Massive crop failures worldwide are caused by abiotic stress. In plants, adverse environmental conditions cause extensive damage to the overall physiology and agronomic yield at various levels. Phytochromes are photosensory phosphoproteins that absorb red (R)/far red (FR) light and play critical roles in different physiological and biochemical responses to light. Considering the role of phytochrome in essential plant developmental processes, genetically manipulating its expression offers a promising approach to crop improvement. Through modulated phytochrome-mediated signalling pathways, plants can become more resistant to environmental stresses by increasing photosynthetic efficiency, antioxidant activity, and expression of genes associated with stress resistance. Plant growth and development in adverse environments can be improved by understanding the roles of phytochromes in stress tolerance characteristics. A comprehensive overview of recent findings regarding the role of phytochromes in modulating abiotic stress by discussing biochemical and molecular aspects of these mechanisms of photoreceptors is offered in this review.

## 1 Introduction

**Fig. 1:**
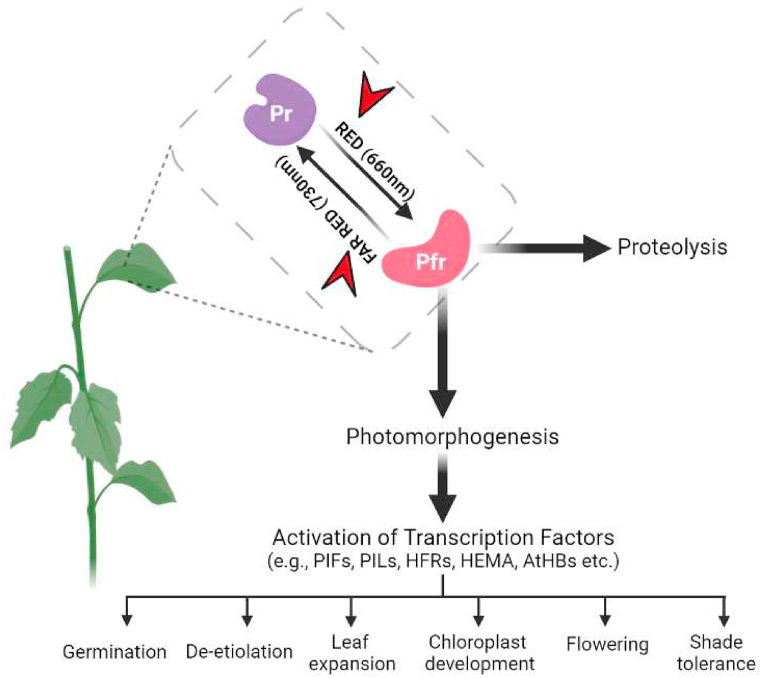
Schematical demonstration of photomorphogenic responses in higher plants. Phytochromes and their transcription factors are shown to mediate photomorphogenic responses to red/far red light. Seed germination, de-etiolation, leaf expansion, chloroplast development, shade tolerance, and flowering are among these responses. Several transcription factors are involved in the photomorphogenesis process, including PHYTOCHROME-INTERACTING FACTOR (PIF); PHYTOCHROME-INTERACTING FACTOR-LIKE (PIL); LONG HYPOCOTYL IN FAR-RED 1 (HFR1); GLUTAMYL-Trna REDUCTASE 1 (HEMA1); and *Arabidopsis thaliana* HD-ZIP protein (AtHBs).

Light is one of the primary environmental signals that control plant growth from germination to flowering [1]. Light perception is triggered through sophisticated photoreceptors systems that sense from UV-B to far-red light wavelengths. Once activated, conformational alterations in the structure of photoreceptors initiate a highly complex signalling cascade from protoplasmic to nucleus, resulting in dramatic changes in the transcriptional profile [[2], [3], [4]]. Four major families of photoreceptors were identified in plants: phytochromes (PHY), associated with red/far-red light wavelength perception, cryptochromes (CRY) and phototropins (PHOT), the conserved blue light photoreceptors (300–500 nm), and the ultraviolet resistance locus 8 (UVR8 - 280–290 nm) [[5], [6], [7], [8]]. While CRYs and PHOTs are key regulators of flowering and stomatal opening [9,10], UVR8 is one of the photoreceptors that regulate the E3 ubiquitin ligase [11] and induced several physiological responses, including hypocotyl length reduction [12]. Early associated as a central component of red(R)/far-red(FR) light perception [13] PHYs family have also been extensively studied as a mediator between the external environment and cell response [14,15]. The biologically inactive red-light absorbing PHY (Pr) remains in the cytosol, once activated by red light, Pfr is converted to the far-red-light absorbing form (Pfr) and is transported to the nucleus upregulating photomorphogenesis-associated genes [16]. The conversion of Pr to Pfr could be reverted by far-red light or dark, where both phytochromes are hence in photostationary equilibrium (Fig. 1). A crucial aspect of Pfr translocation to the nucleus is its interaction with downstream regulators of transcriptional cascades that affect plant growth (Fig. 1). Phytochromes are classified into types I and II in Angiosperms based on physiological and spectroscopic data [11]. Researchers discovered five apoprotein-encoding genes for PHYs in *Arabidopsis thaliana* (PHYA, PHYB, PHYC, PHYD and PHYE) 120 years after they were first discovered. Despite the conserved mechanism of apoprotein binding to its associated chromophore [17], plant species may differ in PHY types due to the duplication of an ancient phytochrome gene [18]. For instance, as opposed to dicots, monocots have three phytochromes: PHYA, PHYB, and PHYC, where single-copy genes have been found in rice (*Oryza sativa*) [19]. As a result of tetraploidization in the original lineage of maize, the three phytochrome genes, PHYA1, PHYA2, PHYB1, and PHYB2, and PHYC1 and PHYC2, have homologous pairings. This indicates that PHYD/E plays a crucial role in dicot phytochrome signalling, yet different from monocot phytochrome signalling [20].

Photoreceptors have been associated with abiotic stress response in plants [[21], [22], [23]]. The term abiotic stress refers to environmental factors that harm living organisms, such as salt, drought, low or high temperatures, and other environmental extremes that contribute to crop loss. Different to plant tolerance for biotic stress, abiotic stress triggers multigenic responses, becoming complex to detect, regulate, and genetic modification. Genetic engineering of light-associated genes has emerged due to their enhanced resistance to abiotic stresses in plants. Here, we discuss recent developments in photomorphogenesis engineering for improved tolerance to abiotic stress in higher plants, focusing on *PIF*, *PHYA*, *PHYB*, and *CRY*-related stress responses.

## 2 Regulation of photomorphogenesis by phytochromes and phytochrome-interacting factors (PIFs)

### 2.1 PHY and PIF molecular signalling pathway

Two distinct stages for plant growth in response to light perception are currently established: skotomorphogenesis (dark-grown plants) and photomorphogenesis (light-grown plants). Plants cultivated in the dark exhibit etiolated characteristics, such as taller hypocotyls, apical hooks, and yellowish cotyledons [24]. In contrast, light-grown plants have small hypocotyls and open and extended cotyledons. Light also impacts growth and development processes, such as shade avoidance, photoperiodic blooming, and directional growth [25].

**Fig. 2:**
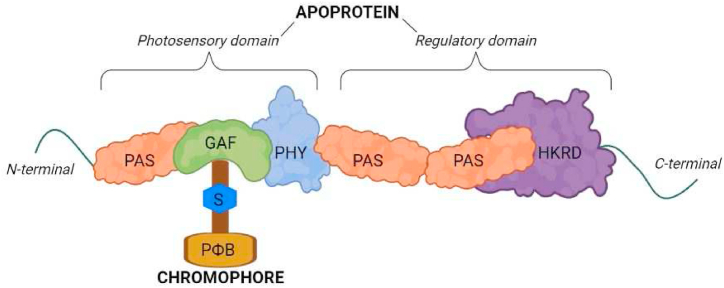
Schematical representation of phytochrome domain structure. The photosensory domain holds the chromophore and includes PAS, GAF and PHY subdomains. As a signal sensor, the PAS domain detects signals, while the GAF domain detects light signals. A chromophore group (PФB) is bound via thioether linkage (S) to a cysteine residue in the GAF subdomain. The phytochrome region is formed by these subunits, which regulate changes in physiology in response to red and far-red light. A more reactive conformation of phytochromobilin (PФB) and its binding pocket is maintained by the PHY domain. Assembling the apophytochrome with PФB takes place in the cytoplasm after PФB is synthesized in the chloroplast. PAS repeats and HKRD subdomains make up the regulatory domain. In phytochrome signalling, the HKRD domain acts as a serine-threonine kinase. Plant phytochrome dimerization and nucleolar organization signals occur in the PAS subdomain. PAS, Per (period circadian protein) - Arnt (aryl hydrocarbon receptor nuclear translocator protein) - Sim (single-minded protein) domain; GAF, cGMP phosphodiesterase - adenylate cyclase - FhlA; HKRD, histidine-kinase-related domain.

Upon light exposure, PHYs primarily respond to the R and FR spectrums. Apoprotein-chromophore conformational interactions (Fig. 2) allosterically change from the inactive Pr to the active Pfr through to the R spectrum perception [19]. PHYs are translocated into the nucleus, where they interact with several light-signalling proteins to downstream control gene transcription [26]. One of the most dramatic repressors of photomorphogenesis in plants is the transcription factor PHYTOCROME-INTERACTING FACTORS (PIF) [27]. PIFs are helix-loop-helix (bHLH) transcription factor superfamily, which in turn acts downstream of PHYs [28], promoting the etiolated skotomorphogenic phenotype of seedlings in darkness. The transcriptional reprogramming promotes to PIFs proteolytic degradation mediated by Pfr PHYs leading to the characteristic photomorphogenic phenotypes [29].

In *Arabidopsis thaliana*, PIF1, PIF3, PIF4, PIF5, PIF6 and PIF7 interact specifically with PHYB via the Active PHYB Binding (APB) domain [30,31]. Additionally, PIF1 and PIF3 interact with PHYA via the Active PHYA Binding (APA) domain [32,33]. Contrary to *Arabidopsis*, *Oryza sativa* (rice), and *Zea mays* (maize) have not been extensively studied for phytochrome-PIF relationships. Only one rice protein (OsPIL15) has an APA motif, despite six PIF-LIKE proteins. OsPHYB, PHYB/PHYC, and PHYC isozymes from the rice PIL family have only been reported to interact *in vitro* with OsPIL14 [34,35], however, both, *Arabidopsis* and rice, have been shown to promote hypocotyl and internode elongation using PIFs and OsPILs [18,35,36]. Regarding maize plants, the Pfr version of ZmPHYB1 interacts with ZmPIF3.1, but not with ZmPHYB2. There is no interaction between them and any other ZmPIF [26,37] ZmPIFs have not yet been demonstrated to be involved in light signalling. Although phytochrome-PIF signalling mechanisms in *Arabidopsis thaliana* have been extensively investigated [38], little is known about phytochrome-PIF signalling in other terrestrial plants.

The role of PIFs in various biological processes has been studied extensively by genetic, biochemistry, and physiological approaches. Thus, the genetic manipulation of *PIF*s has inherent photomorphogenic properties involved with biochemical reactions, circadian clock control, thermomorphogenesis, and hormone signalling [25].

### 2.2 Different PIF proteins control several aspects of plant development

Several target genes and physiological responses mediated by specific isoforms of PIFs proteins have been investigated The PIF1 protein, for instance, suppresses light-dependent seed germination. One of the most critical seed germination regulators, LEUNIG HOMOLOG, interacts with PIF1 and regulates abscisic acid (ABA)- and gibberellic acid (GA)-related gene expression [18,39]. The PIF1 gene family also plays essential roles in the biosynthesis of chlorophyll and the development of plastids [40]. In contrast to other PIFs, PIF2 regulates seedling de-etiolation in response to blue, R, and FR lights. Additionally, PIF2 inhibits gene expression by interacting with PIFs 1, 3, 4, and 5 [19]. PIF2 also forms heterodimers with HFR enhancing photomorphogenesis by a mechanism that remains unclear [41]. Furthermore, PIF2 is downstream of the intrinsic regulation mediated by the interaction between PHYB2 and CONSTITUTIVE PHOTOMORPHOGENESIS 1 (COP1), which enhances and inhibits the stability of the protein, respectively [19]. The abundance of PHYB levels is primarily controlled by PIF3 during seedling de-etiolation. Additionally, Zhang et al. [42] demonstrated that PIF3 regulates hypocotyl elongation in light through inhibition of *C-REPEAT BINDING FACTOR* (*CBF*) expression. Additionally, PIF3 presents a similar role to PIF1 in the impairment of chlorophyll biosynthesis and photosynthesis [43]. PIF is also involved with an epigenetic mechanism by HISTONE DEACETYLASE15, which repress gene expression by decreasing histone acetylation [44].

Hypocotyl elongation is regulated by PIF4 in response to environmental conditions such as light, shade, temperature, and circadian cycle [45]. The promoter regions of auxin biosynthesis genes and other targets are activated by binding to them. The constitutive activation of PIF4 promotes hypocotyl hyper elongation resulting in a physiological imbalance through increasing auxin contents [46,47]. Hypocotyl lengthening by PIF4 under varied circumstances provides overall fitness and survival advantages. Plants use a variety of regulatory mechanisms to prevent such events, including reducing PIF4 transcriptional activity. The interaction between EARLY FLOWERING3 and PIF4 results in PIF4 activity being reduced in a manner that is not dependent on the evening complex [48].

There are several pathways in which PIF5 and PIF4 crosstalk in molecular physiology regulation. Plants use PIF5 and PIF4 as regulators of chlorophyll degradation and shade avoidance [49,50]. Two splice variants of PIF6 control seed dormancy. When PIF6 is overexpressed, hypocotyl elongation is inhibited in continuous R light, contrary to the usual role of PIFs as negative photomorphogenesis regulators [51]. While its functional significance remains unclear, the fact that PIF6 interacts with TOC1 is intriguing.

When seedlings are exposed to R light, PIF7 inhibits de-etiolation [52]. PIF7, PIF3, and PIF4 are thought to promote the elongation of hypocotyls in plants that receive continuous R light. Auxin biosynthesis genes are also controlled directly by PIF7, allowing them to govern shade avoidance responses [53]. Furthermore, PIF7 and PIF4 downregulate *CBF* gene expression in response to photoperiod [54]. Despite the limited number of studies conducted to characterize the role of PIF8, Oh et al. (2020) discovered significant accumulations of PIF8 in far-red light compared to darkness. Additionally, they observed an interaction between PIF8 and the Pfr form of PHYB and PHYA, albeit weakly. The overall interaction between PIF8 and PHYA plays a crucial role in seed germination, hypocotyl elongation, and orientation. Further research in this area could provide valuable insights into the regulatory mechanisms governing these processes [55].

## 3 Phytochrome engineering for improved tolerance under various abiotic stress conditions

### 3.1 Salt stress

A growing concern in agriculture is soil salinity, by which plant growth is adversely impacted due to the impartment in water and nutrient absorption. Salt stress triggers complex downstream signalling cascades in plants, which are potential targets for genetic manipulation to increase salt tolerance in crops. McElwain et al. [56] identified phytochromes as critical factors in salt stress responses, for instance, when plants are simultaneously exposed to several ratios of R/FR lights and salt stress, the response of photosynthesis performances presented drastic changes due to the control of the levels of reactive oxygen species (ROS) [16]. Salt stress has earlier established to increase ROS accumulation in plant tissue. Prolonged stress increases antioxidant enzymes, such as catalase (CAT), ascorbate peroxidase (APX), superoxide dismutase (SOD), glutathione reductase (GR), guaiacol peroxidase (GPOX), and peroxidase (POD) [57]. As a result, ROS and antioxidant enzymes can serve as markers of plant tolerance to stress. Plant cells are damaged by ROS through lipid peroxidation in membranes. Plants synthesize ROS scavengers as a protection mechanism against high salinity conditions. Heme oxygenase (HO) is well known among mammals and plants as an antioxidant enzyme. As a result of oxygenating heme, it produces Fe^2+^, CO, and biliverdin as byproducts [58]. *Pisum sativum* plants lacking *phytochrome chromophore* (*pcd1*) failed to convert heme to biliverdin, indicating an interaction between PHYs and HO, and the mechanism of light signalling components in response to salt stress [59].

Although it remains unclear the role of specific PHYs in these effects, understanding how PHYs modulate ROS scavengers, such as GPOX and SOD is essential for further manipulation of cultivar resistance to salt stress. *Hy1* mutants showed long hypocotyls and were completely insensitive to R/FR in *Arabidopsis*, demonstrating a mutually reinforcing relationship between PHY and HO [60]. PHY and HO crosstalk boosts the antioxidant system, which improves salt tolerance in plants. Furthermore, the signalling mechanisms mediated by PHY affect the expression of genes that confer salt tolerance. Transgenic *Arabidopsis* plants overexpressing the salt tolerance protein STO developed roots quicker than plants under salt stress treatment [61]. It has been shown that STO is suppressed by the COP1 and SPA1-COP1-PIF1 kinase regulatory complex (PIF1), which is degraded via proteasome 26S by PHYB in response to red light [61]. A negative role of PHYB in salt stress has also been indicated in several reports. Salt stress reduced malondialdehyde (MDA) levels in *Solanum lycopersicum* (tomato) *phyB1* mutant, indicating a decrease in oxidative damage [62]. reported enhanced ROS-scavenging activity (CAT, POD, and SOD) and reduced ROS content when mimicked by *phyB1* mutants by increasing the inactive *phyB1*-form content. Remodelling of the oxidative stress mechanism is not the only factor involved in the role of PHYB in salt tolerance improvement. Salt tolerance is induced by several proteins other than PHYB through different mechanisms [61]. By preventing the buildup of toxic Na^+^ concentrations and improving salt tolerance, HKTs, high-affinity K^+^ transporters, have been found to prevent Na^+^ toxic concentrations [19]. The expression of putative Os*HKT* genes is suppressed in rice *phyB* mutants when plants are grown in low Na ^+^ concentrations [63]. As a result of this regulation, salt tolerance is induced in plants. Another side, salt stress could disrupt the photosynthetic system, tomato *phyAB* mutant plants presented higher maximal quantum efficiency of PSII and quantum efficiency of PSII (photosynthesis performance indicators) than the wild-type counterpart when both are cultivated under salt stress conditions [62]. Remarkably, the abundance of salt at high concentrations has been found to significantly alter the transcriptional profile of *Phaseolus vulgaris*, leading to an upregulation in the transcript levels of three specific PIF genes: Pvul-PIF-3.3, Pvul-PIF-4.1, and Pvul-PIF-4.2. These three PIF genes are part of the five PIF genes present in the common bean's genome. This discovery suggests that the modulation of these PIF genes could play a vital role in the response of *Phaseolus vulgaris* to salt stress [64].

These pieces of evidence indicated that, despite the low ratio of R/FR light increased photosynthesis performance, the possible pleiotropic effects from the double *phyAB* mutant represent positive regulation of photosynthesis in plants under high salinity conditions. As evidenced in Table 1, these views suggest new points for studying salt stress response through the regulation of the PHY-associated signalling pathway in different plant species. Altogether, these studies open a promising alternative to manipulation at an upstream level and improve the resistance of salt stress in simultaneous ways.

**Table 1:** Summary of recent studies on the effects of PHY and PIF mutations on plant salinity tolerance.

Plant	Phy/PIF^a^	Expression	Observations	Reference
*Nicotiana tabacum*	PHYA, PHYB	Single and double mutants	Improved salinity stress tolerance with lower electrolyte leakage and MDA concentration, improved antioxidant system, and increased ABA and JA content	[65]
*Oryza sativa*	PHYB	Mutant	Enhanced salt tolerance, improved Na + to K+ ratio, higher cell membrane integrity	[63]
*Zoysia japonica*	PHYA	Serine 599 Alanine hyperactive PhyA mutant	Improved salt tolerance, higher chlorophyll content, lower hydrogen peroxide level, higher proline accumulation, and improved photosynthesis	[66]
*Agrostis stolonifera*	PHYA	Serine 599 Alanine hyperactive PhyA mutant	Improved salt tolerance, higher chlorophyll content, lower hydrogen peroxide level, higher proline accumulation, and improved photosynthesis	[66]
*Arabidopsis thaliana*	MfPIF8	Overexpressed	Improved salt tolerance	[67]
*Capsicum annuum*	PIF8	Mutant	higher sensitivity to salt stress	[68]
*Arabidopsis thaliana*	MfPIF1	mutant, overexpressed	Improved salt tolerance	[69]

a Phy – phytochrome, PIF – Phytochrome interacting factor.

### 3.2 Drought stress

Global climate change has made drought stress increasingly problematic for crops. Plants undergo morphological, physiological, and molecular changes in response to drought. In drought-tolerant plants, early flowering and rapid transpiration are indicators of evasive behaviour [70]. Phytochromes and PIFs play a crucial role in stress responses in plants at the physiological level, including changes to the stomatal aperture, growth regulation, ROS levels, alterations in osmoprotectants, and sensitivity to ABA [62],[71]],[72]. PIFs and PHYs may regulate stress-induced gene expression at the molecular level [61]. Plant PHYs and PIFs have been studied extensively for their effects on drought tolerance [73]. The molecular mechanisms of drought tolerance in plants have been studied in several previous research studies [reviewed in 19, 49, 72]. Some findings have led to doubts regarding a connection between phytochromes and drought stress because PHYs regulate leaf transpiration. Also, phytochromes could influence how relatively dormant seeds germinate under drought stress [40,74,75]. The results from studies focusing on the role of phytochromes and PIFs identified in recent years are summarized in Table 2.

**Table 2:** Summary of recent studies on the effects of PHY and PIF mutations on drought tolerance in plants.

Plant	Phy/PIF^a^	Expression	Observations	Reference
*Triticum aestivum*	PHYA	Drought-tolerant cultivars	Putative *phyA* genes upregulated under drought conditions	[76]
*Solanum lycopersicum*	PHYA	mutant, deficient	Less tolerance to drought stress	[62]
*Solanum lycopersicum*	PHYB	mutant, deficient	Increased pigment content in leaves, osmoprotectant levels, and growth. Lower MDA levels. Improved tolerance to drought stress	[62]
*Solanum lycopersicum*	PHYA, B1, B2, E, F	mutant, deficient	Lower proline content under drought conditions	[77]
*Oryza sativa*	PHYB	mutant, deficient	Increased peroxide in roots, increased antioxidant activity. Improved tolerance to drought stress	[70]
*Oryza sativa*	PHYB	mutant, deficient	Impaired drought escape response, earlier flowering under drought conditions	[78]
*Oryza sativa*	PHYB	mutant, deficient	Improved tolerance to drought stress.	[79]
*Zea mays*	PIF1	mutant, overexpressed	Reduced transpiration. Improved tolerance to drought stress	[80]
*Zea mays*	PIF1	mutant, overexpressed	Reduced stomatal aperture, increased ABA sensitivity. Improved tolerance to drought stress	[81]
*Zea mays*	PIF1, 3, 4, 6	wild type	Upregulated during PEG stress exposure indicates a role in drought tolerance.	[82]
*Arabidopsis thaliana*	PIF3	overexpressed	Improved drought tolerance, lower MDA levels, and increased ABA accumulation	[83]
*Arabidopsis thaliana*	PIF1	mutant, overexpressed	Improved drought tolerance	[67]
*Arabidopsis thaliana*	PIF8	overexpressed	Improved drought tolerance	[69]
*Malus domestica*	PIF3	mutant, overexpressed	Enhanced drought resistance in apple callus.	[84]
*Arabidopsis*	PIF3	mutant, overexpressed	Enhanced drought resistance.	[84]
*Arabidopsis thaliana*	PIL1	mutant, overexpressed	Improved drought tolerance when co-overexpressed with DREB1A.	[83]

a Phy – phytochrome, PIF – Phytochrome interacting factor.

It has been shown in some studies that physiological adaptations to drought in *Nicotiana tabacum* and *Arabidopsis* mutants are influenced by PHY-chromophores. On another side, ABA, a plant hormone that acts as a negative regulator of stomatal conductance, also contributes to avoiding leaf water loss [62]. It is suggested in several reports that PHYB negatively affects stomatal conductance under drought stress [45,63,85]. As a result of drought stress, *Arabidopsis phyB* mutants showed lower stomatal conductance than WT thought stimulation of ABA production, perception, and signalling [85]. *phyB* mutation impairs the stimuli of key components present in the ABA signalling cellular pathway, such as ABCG22 (ATP binding cassette) and PYL5, decreasing drought resistance and water loss prevention via stomatal closure [86]. The inhibition of stomatal opening results in photosynthesis impairment *Arabidopsis phyB* mutants presents reduced transpiration rates, resulting in decreased CO_2_ absorption and photosynthesis impairment due to the reduction of stomatal opening and density in the leaf [87]. Although the decrease in water loss seems favourable, plants exposed to high levels of active photosynthetic radiation may be threatened by reduced CO_2_ absorption. Excess excitation energy may be converted to ROS if the fluorescence or heat is not dissipated correctly due to low CO_2_ concentrations. Based on these findings, mutants of *phyB* are more susceptible to drought stress. While *phyB* has been implicated in drought stress responses in certain studies, it also seems to be involved in antioxidant signalling [88]. A favourable consequence of drought stress on rice *phyB* mutant genotypes was the overexpression of *APX*s and *CAT*s, which resulted in drought tolerance in these plants [63]. These elucidations may explain how rice phyB mutants can maintain crop production despite lower photosynthetic rates, once in drought-stressed *phyB* plants, stomatal density and net CO_2_ absorption decreased, but rice yield was not affected. The *PHYB* gene also modulates drought tolerance in tomato plants, particularly in *phyB1* mutants. *phyB1* plants demonstrated longer shoots and roots and high amounts of proline, glycine betaine, and phenylalanine concentrations in their tissues than WT plants [89]. The osmoprotectants proline and glycine-betaine have been found to boost cellular osmotic control in drought-resistant plants [[89], [90], [91]]. Under the escape route above, phytochromes function as drought stress mediators.

The signalling pathways of ABA and PHYs communicate under dry conditions. It has been demonstrated that PHYs inhibit the metabolism of ABA by suppressing biosynthesis and signalling-related genes. ABA levels were higher in *Nicotiana plumbaginifolia pewl* mutant compared to WT during dehydration tests, and, consequently, water retention remains higher in this adverse situation [92]. Plant tissues, especially leave, require osmoprotectants to maintain water homeostasis during water-stress conditions [70,93]. Under drought conditions, phytochromobillin-deficient tomato plants (*au* mutants) exhibited lower levels of the osmoprotectant proline, indicating that PHYs play a critical role in proline accumulation [77]. In tomatoes, PHYA regulates root growth, osmoprotectant accumulation and stress indicators levels in *phyA* plants exposed to water stress [63]. Furthermore, the gene expression profiling of *Triticum aestivum* plants revealed two putative *PHYA* upregulated in drought-tolerant cultivars [76], alluding to the key role of PHYA in the ability of wheat to tolerate drought conditions. Overall, PHYA plays an essential role in drought tolerance in plants.

Plants deficient in PHYB have been shown to have better drought response than their WT counterparts in different plants. In *Solanum lycopersicum*, PHYB acts as a negative regulator for growth, pigment biosynthesis (chlorophyll, carotenoids, etc.), and osmoprotectant accumulation during stress [62]. In *Orzea sativa*, *PHYB*B deficiency resulted in higher levels of hydrogen peroxide in the roots, despite the increased antioxidant activity. This may point to plants using hydrogen peroxide as a potential secondary messenger to activate the ROS removal pathway. Overall, *phyB* deficiency resulted in better drought tolerance [78].

Conversely, *phyB* mutation resulted in earlier flowering and impairment of the drought escape response in rice. The drought escape response enables plants to shorten their life cycle and earlier produce seeds to further avoid more severe drought conditions [79]. Since a deficiency in PHYB also results in improved drought tolerance, the drought escape response is possibly unnecessary. Thus, the impairment is not as severe. Furthermore, PHYB regulates various responses to drought conditions, including stomatal aperture, homeostasis, and oxidative stress.

Increasing the complexity of PHY-drought-related responses, the main role of PIF proteins also have been established. In *Zea mays*, the overexpression of *PIF1* resulted in increased drought tolerance through reduced transpiration-mediated inhibition of stomatal opening and increased ABA sensitivity [80,81]. Moreover, the four Zm*PIF*s were upregulated when plants were exposed to 20% polyethylene glycol (PEG) stress, indicating that they play a role in the response of plants to drought or water-limiting conditions [82]. Interesting findings have emerged from the study of drought treatment effects on Nicotiana tabacum. Notably, the transcriptional levels of PIF1 were significantly upregulated under drought conditions. Surprisingly, contrary to the findings observed in maize, the induced knockout of this gene resulted in improved drought tolerance in tobacco plants. The knockout of PIF1 led to enhancements in various drought adaptive traits, including increased osmotic adjustment, elevated antioxidant activity, and improved photosynthetic efficiency. Moreover, the reduction in water loss rate was attributed to decreased ABA content in the tobacco plants with the knockout *PIF1* gene [94].

Interestingly, it was demonstrated that the overexpression of carrot PIF3 in Arabidopsis led to enhanced drought tolerance with higher antioxidant capacity, lower MDA content, and increased accumulation of ABA [83]. It has been shown that *Arabidopsis* can cope better with droughts when exogenous PIFs are expressed [38,[83], [84], [95]]. In *Arabidopsis*, the expression of PIF1 from *Myrothamnus flabellifolia* (also called the resurrection plant due to its desiccation tolerance) resulted in increased drought tolerance as a consequence of osmoprotectants, and antioxidant enzyme activity enhanced, and reduction in transpiration by a decrease in stomatal aperture [67]. More recently, PIF8 from, *M. flabellifolia* was overexpressed in *Arabidopsis,* and improvement of drought tolerance was reported at the seedling and adult stages. The improved drought tolerance was accompanied by an increase in primary root length, stomatal aperture, decreased water loss rate, and higher chlorophyll content [69]. Overexpressing OsPIL1, a homolog of PIF4, and dehydration-responsive element-binding 1A (DREB1A), also promoted drought tolerance in *Arabidopsis* and other crops by binding dehydration-responsive elements and upregulating stress-induced gene expression [38]. Interestingly, the delayed flowering seen from the overexpression of DREB1A in *Arabidopsis* was facilitated by the coexpression of OsPIL1. It was indicated by these findings that plants can be modified by combining more than one strategy to improve drought tolerance and potentially mitigate the adverse effects of overexpression of either factor.

Overall, it has been shown in recent studies that plant drought tolerance increases when PIFs are overexpressed. Combined manipulation of *PHYA* and *PIF*s protein emerged as a promising strategy to improve plants against drought stress by triggering important physiological modifications such as the reduction of transpiration and changes in the stomatal aperture. It also has demonstrated that exogenous *PIF*s expression presents the potential to produce drought-tolerant transgenic plants.

### 3.3 High- and low-temperature stress

Plants are susceptible to temperature fluctuations. Plant species exhibit a wide range of temperature tolerances by natural strategies to detect temperature swings in the environment and initiate germination, ensuring ideal growing conditions for seedlings. Although the perception of temperature in plant development is critical for survival, the identity and mechanisms of thermo-sensors in plants are still poorly understood [96]. It has been recently demonstrated that PHYs are involved in temperature perception as well, as reported for *Arabidopsis thaliana* seedlings growing under different temperature regimes [23,97]. Moreover, plants carrying a constitutive activated PHYB mutation, display inhibition of warm-associated gene expression independently of temperature gradient [23]. Interestingly, the functional substitution of PHY isoforms according to a temperature gradient was observed: *phyB* mutant plays an essential role in *Arabidopsis* early flowering at 22 °C, though this phenotype was abolished at 16 °C, while *phyA* mutant presented a similar flowering time than the wild type in both temperature. Additionally, the effects of *phyD* deficiency in *phyAphyB* background display an extremely early flowering phenotype at 22 °C without effects at 16 °C compared to the wild type. *Arabidopsis* flowered considerably earlier at 16 °C only when the quadruple mutant *phyAphyBphyDphyE* grew at this temperature [21]. Altogether, these results demonstrate that PHYB and PHYD have higher action at 22 °C, PHYE is most important at 16 °C and PHYA presented a similar effect in all biological temperatures, suggesting an attractive thermo-sensor specificity role for each PHY. In *Arabidopsis*, PHYA, PHYB, and PHYE also have been studied for their roles in responding to cold and warm temperature signals during seed germination [19,98,99]. When seeds react to temperature, they can time their germination with optimal conditions for seedling establishment, avoiding stressful situations and ensuring successful seedling establishment. According to Song et al. [100], *Arabidopsis* has many heat-responsive genes incorporated into the light- and phytohormone-mediated pathways. In the light-dependent stress response, PHYB acts like an on/off switch to control several other genes. Furthermore, the importance of PHYB in temperature perception and response was confirmed by morphological studies of *phyB* mutants [100]. Shade reduces tissue temperature. Thus, the likelihood of heat shocks is due to the light quality signals of neighbouring vegetation. PHYB activity is diminished by neighbour signals, leading to an increase in PIF abundance, according to Arico et al. [98]. Heat stress tolerance was high for the *phyB* mutant even when subjected to simulated sunlight, but low for the PIF multiple mutants when subjected to simulated shade [98]. Moreover, it has been demonstrated that the double mutants *phyB1phyB2* in tomato plants induce a temperature-insensitive phenotype during de biosynthesis of chlorophyll in leaves and carotenoids and fruits [101], and prevent cell membrane injuries and water loss [102]. Interestingly, the tomato *phyA* mutant has been related to the improvement of levels of osmoprotectants when plants are grown under heat stress [102]. The data summarized in Table 3 represents the PHY-associated responses mediated by temperature perception in plants described in different plant species. Under heat or cold stress, the cellular viability of plants is significantly affected by the quality of light they receive.

**Table 3:** Summary of recent studies on the effects of phytochrome and PIF mutations on high/low-temperature stress tolerance in plants.

Plant	Phy/PIF^a^	Expression	Observations	Reference
*Solanum lycopersicum*	PHYA, PHYB1B2	Mutants	Enhanced heat tolerance	[101,102]
*Arabidopsis thaliana*	PHYB, PIF	Mutants	Enhanced heat tolerance in *phyB* mutants and low heat tolerance under simulated shade in *pif* multiple mutants	[103]
*Arabidopsis thaliana*	PHYB	Mutants	Enhanced heat stress tolerance	[104]
*Arabidopsis thaliana*	PIF4, PIF5	Overexpression	Promotion of heat stress-induced leaf senescence	[50]
*Solanum lycopersicum*	PIF8	Overexpression	Increased cold tolerance	[105]
*Poncirus trifoliata*	PIF8	Virus-induced gene silencing mediated suppression of PIF8	Increased cold sensitivity	[105]
*Capsicum annuum*	PIF8	Mutant	Higher sensitivity to cold stress	[68]

a PHY – phytochrome, PIF – Phytochrome interacting factor.

During temperature acclimation, damage to the thylakoid membrane produces ROS [16,106]. As a result of physiological and morphological changes mediated by PHYB, both abiotic and biotic stresses can be alleviated in stressed plants [19,45]. In-depth studies of a broad range of plant species need to be conducted to understand how PHYB impacts plant responses to high light and heat/cold stresses. When developing plants that can withstand various environmental challenges, the PHYB photoreceptor may be one of the molecules to consider. There is still a need for studies to demonstrate that PHYB proteins play a consistent role in increasing plant resistance to environmental stress [107]. The application of genetic modification to overexpression of *PHY*s-associated genes or mutation in the PHY protein structure that inhibits the impact of temperature in the conversion from Pfr to Pr has been developed in *Arabidopsis*, *Avena sativa* and tomato as promising future strategies for plant improvement [108,109].

The role of PHYs in heat stress tolerance has been investigated in many studies, but the underlying molecular networks are still poorly understood. In *Arabidopsis*, the PIF4 and PIF5 proteins appeared to promote heat stress-induced leaf senescence. Multiple biological processes, including auxin signalling pathways, have been implicated in the function of PIF4 and PIF5 [50]. PIF4 has largely been associated with *Arabidopsis* and tomato as a key component that integrates temperature and PHYB signalling with differential gene expression during the hypocotyl elongation process [103,104]. The PIF4 stabilization under high temperatures promotes the enhancement of auxin-biosynthesis gene expression triggering a similar phenotype to that of dark-grown plants [110]. Furthermore, PIF7 also has been recently demonstrated for early response to high temperatures in *Arabidopsis* [111].

PHYs and PIFs have also been demonstrated in several studies to play a role in cold stress tolerance. Transgenic tomato plants and *Citrus grandis* (grapefruit) callus overexpressing CsPIF8 were colder tolerant. Conversely, the suppression of PIF8 increased cold sensitivity in *Poncirus trifoliata* seedlings [105]. Similarly, CaPIF8-silenced pepper plants also showed enhanced sensitivity to cold, increased electrolyte leakage, and altered stress-related gene expression. Additionally, it was demonstrated that CaPIF8 regulates the cold response by promoting the expression of the C-repeat binding factor 1 (CBF1) gene [68].

### 3.4 High-light (HL) stress and ultraviolet B radiation (UV–B)

The growth and development of plants are heavily influenced by light intensity. Adaptation responses, cellular damage, and, ultimately, plant death can result from adverse intensity, range, and duration of light. Several efficient protective mechanisms have evolved in plants that enable them to survive in unfavourable light conditions. The xanthophyll cycle, electron transport chain, and photorespiration pathway are primarily involved in these mechanisms [112]. As a result of HL stress, ROS are produced at elevated levels. ROS molecules hamper the photodamaged PSII repair cycle in higher plants [16]. Complex crosstalk between photosensory molecules at the cellular level prevents excessive light from damaging cells. It has been suggested that phytochrome signalling regulates plants' sensitivity to radiation damage [19]. Researchers have uncovered some of the biochemical and molecular processes behind this phenomenon over the last 40 years. The “ROS wave” of a plant cell, described as the generation of ROS from ROS, induces systemic stress memory. However, within 3 h of its activation, stomata become insensitive to ROS promoting the opening [113]. In recent studies, PHYB emerged as crucial to triggering ROS waves and systemic and local stomatal aperture closure responses in response to HL stress [113]. According to another recent study, ROS production, transcript expression, and plant acclimation to HL stress also are regulated by PHYB. Finally, PHYB is a principal component that avoids photosynthetic damage in plants under UV and high light conditions [114].

Interestingly, PHYB regulates ROS production during stress, even in a Pr form into the cytosol. Furthermore, along with respiratory burst oxidase homolog proteins, PHYB modulates thousands of transcripts under HL stress [115]. *Arabidopsis* HL-responsive genes were identified by Huang et al. [116] using comprehensive transcriptome analysis. The dynamic regulation of genes involved in photosynthesis, ABA, and the phenylpropanoid pathway was responsible for the response of plants to HL. Additionally, PIF genes and blue/UV-A photoreceptors were also affected by HL. PIFs have been shown in recent studies to prevent photooxidation during the transition from skotomorphogenesis to photomorphogenesis in *Arabidopsis* [82,117]. A limited number of molecules, including kinases and hormones, may interact with PIFs in phytochrome signalling.

Moreover, PIFs are crucial to regulating photomorphogenesis in seedlings, mainly by stimulating the expression of protochlorophyllide reductases (*POR*s), enzymes that convert protochlorophyllide to chlorophyll (Fig. 1). During photomorphogenesis, protochlorophyllide accumulation causes photo-oxidative damage. Therefore, the seeds of the *pif1* mutant are more sensitive to light than those of non-mutant seeds [118]. The effects of PHYA, PHYB and PIF in the physiological aspects of plants under high-light stress are summarized in Table 4. Altogether, it represents key components as strategies to improve stomatal dynamic, photosynthesis efficiency and ROS control in plants exposed to high light intensities.

**Table 4:** Summary of recent studies on the effects of PHY and PIF mutations on HL and UV-B radiation tolerance in plants.

Plant	PHY/PIF^a^	Expression	Observations	Reference
*Arabidopsis thaliana*	PHYA, PHYB	*phyA, phyB* single mutants, and *phyAphyB* double mutants	PhyB regulates c stomatal aperture closure responses under HL stress	[113]
*Arabidopsis thaliana*	PHYA, PHYB	Mutants	Reduced photosynthetic efficiency in *phyB* plants under UV and HL stresses	[114]
*Arabidopsis thaliana*	PHYA, PHYB	*phyAphyB* double mutants	Reduced PSII efficiency and increased H2O2 content under UV stress	[119,120]
*Solanum lycopersicum*	PHYA, PHYB, CRY1	single (*phyB2, phyB1, phyA and cry1*), double (*phyB1B2, phyAB2 and phyAB1*) and triple (*phyAB1B2 and cry1phyAB1*) mutants	Low resistance to HL in *cry1phyAB1*	[121]

a PHY – phytochrome, CRY – cryptochrome.

## 4 Cryptochrome-related abiotic stress responses in plants

In addition to phytochromes, other light-sensing molecules are becoming increasingly prominent [122]. In combination with blue, green, and UV-A light, CRYs have been shown to affect plant germination and growth. Cryptochrome C-terminal extension (CCE) and photolyase homology-related (PHR) apoproteins are found in various species, from bacteria to humans. Chromophore-binding enzymes have noncovalent binding sites for the two chromophores, methyltetrahydrofolate (MTHF) and flavin adenine dinucleotide (FAD). Because FAD is a necessary enzymatic cofactor for the FAD chromophore of BL, the MTHF chromophore is used in the UV-A region. Three *CRY* genes are present in the *Arabidopsis* genome, two belonging to the plant *CRY* subfamily (*CRY1* and *CRY2*) and one belonging to the CRY-DASH subfamily (CRY3) [123]. CRY1 and CRY2 are associated with various processes in plants, including growth and development. The circadian clock, photoperiod-dependent flowering, and other processes are all controlled by CRY2, whereas hypocotyl elongation and anthocyanin accumulation are inhibited by the same photoreceptor. In chloroplasts and mitochondria, where the DASH protein CRY3 may be present, UV exposure damages DNA [45].

Plant growth is significantly affected by drought in agricultural systems As we understand how plants respond to drought stress, it is not surprising that Crys are critical. It has been shown that CRYs play an essential role in *Arabidopsis* drought stress resistance [124]. *cry1cry2* mutants plants presented drought resistance after 7d of interruption of watering [125]. The interaction of CRYs with COP1 also inhibits stomatal opening through the regulation of downstream signalling components. Due to the interplay between several variables, it is unclear how CRYs control water loss during drought stress. Among these variables are hormones (*e.g.* ABA), and interactions with the phototropin associated with BL perception [124]. Water stress triggers the release of ABA, a hormone that participates in CRY signalling. *Arabidopsis* overexpressing Ta*CRY1a* and Ta*CRY2* of *Triticum aestivum* were less sensitive to osmotic stress and exogenous ABA treatment during germination and post-germination development. The transgenic lines also expressed ABA/stress-responsive genes, including *RD29A* and *ADH1*. Plants overexpressing Ta*CRY1a* were susceptible to osmotic stress, showing a strong reduction in *RD29A* expression and lower levels of ADH1 than those overexpressing Ta*CRY2*.

The effects of temperature stress on protein stability and enzymatic processes in plants are well known. Furthermore, many of the responses to heat stress are mediated by downstream heat shock proteins (HSPs). The transcriptional profile of *HSP*s is heavily influenced by CRYs. *CRY1* suppresses auxin production in *Arabidopsis* seedlings in response to heat stress [126]. CRY1 also binds with the promoter of *IAA19* and *IAA29* in high-temperature conditions through PIF4 interaction [128]. Due to their intricate interactions with other photoreceptors and signalling molecules, BL photoreceptors also play an essential role in low-temperature tolerance. It can be concluded from previous studies that the control of temperature responses mediated by CRYs is still uncommon. Hence, more research in this field needs to be made to establish the components that interact with CRY in response to environmental stress.

## 5 Conclusion and future Directions

**Fig. 3:**
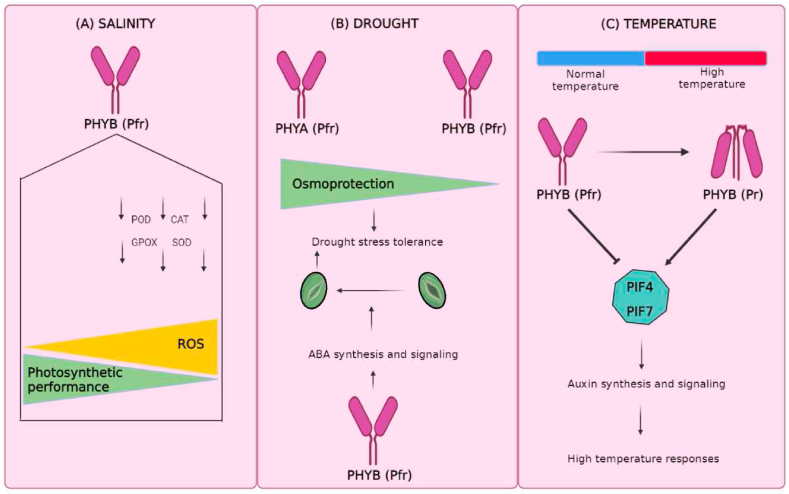
Complex and shifting interaction of PHYs under abiotic stress. (A) PHYB negatively impact plant photosynthesis during salt stress conditions through the inhibition of enzymes associated with antioxidant protection. (B) PHYA and PHYB present, respectively, positive and negative roles in the accumulation of components associated with osmotic protection during drought stress, on another side, PHYB decreases the response associated with drought stress through the reduction in stomatal conductance via ABA synthesis and signalling. (C) High temperature promotes the balance of the PHYB inactive Pr form. The inactivation of PHYB led to PIF4 and PIF7 accumulation which increases auxin synthesis promoting plant phenotype associated with a warm environment.

As sessile organisms, plants have devised innovative strategies for successfully monitory environmental pressures to enable adaptation and survival. Although the complete mechanism of perception and response from stress factors needs to be addressed before moving forward in plant improvement strategies, the role of photomorphogenic-associates proteins in the face to mitigation of abiotic stress is an important piece of knowledge necessary to provide crops and other plants with a multi-stress tolerance. Phytochromes and PIFs have been extensively studied for their critical roles in photomorphogenesis. However, their roles in various abiotic stress conditions are still poorly understood. Stress tolerance has been successfully increased by overexpression of genes associated with metabolites and hormones biosynthesis and antioxidant enzymes, yet adverse effects on plant development were detected. Therefore, it is imperative to explore upstream targets that simultaneously control a myriad of physiological and biochemical parameters at the same time to increase crop productivity and stress tolerance. PHYs play a critical role in controlling plant growth processes and agronomic properties. However, the molecular mechanisms underlying PHY-mediated stress responses in crops remain elusive. Researchers will need a comprehensive understanding of these underlying molecular mechanisms to improve resistance to adverse environmental conditions. In achieving these objectives, novel technologies, such as CRISPR/Cas9 gene editing, could prove invaluable, as they offer highly efficient and precise targeting of candidate genes. To avoid pleiotropic effects resulting from the manipulation of PHYs, new strategies have been addressed with promising results by genetic manipulation applying tissue-specific or inducible promotors. Furthermore, the close association of PHYs with plant photosynthesis (*e.g.* pigments biosynthesis, expression of genes associated with the Calvin-Benson cycle, stomatal opening and chloroplast biogenesis), improved the knowledge regarding PHYs control of photosynthesis parameters under stressful conditions is equally important to improve crop in the scenario of global climate changes. Researchers should pay particular attention to the altered expression of various PHYs and associated factors (*e.g.*, high light, high-temperature stress, or both) that directly deteriorate the PSII machinery. It has been reported that the enhanced accumulation of Pfr form under high-light conditions influences the antioxidant system by increasing the accumulation of carotenoids and flavonoids. An intricate regulatory network involving PHYA and PHYB is responsible for targetting differential gene expression, and biochemical and metabolic changes in the face to regulate physiological processes throughout plant life under more severe abiotic stress (Fig. 3): salinity (Fig. 3A), drought (Fig. 3B) and temperature (Fig. 3C). Photo-biotechnology-driven crop improvement programs could be facilitated by a better understanding of these molecular switches.

## Author contribution statement

All authors listed have significantly contributed to the development and the writing of this article.

## Data availability statement

No data was used for the research described in the article.

## Additional information

No additional information is available for this paper.

## Declaration of competing interest

The authors declare the following financial interests/personal relationships which may be considered as potential competing interests:

MAYANK ANAND GURURANI reports financial support was provided by UAE University College of Science. MAYANK ANAND GURURANI reports a relationship with United Arab Emirates University that includes: employment.
